# Resident Microbiome of Kidney Tumors

**DOI:** 10.3389/or.2024.1393664

**Published:** 2024-05-21

**Authors:** Olga Kovaleva, Polina Podlesnaya, Alexei Gratchev

**Affiliations:** N. N. Blokhin National Medical Research Center of Oncology, Moscow, Russia

**Keywords:** kidney cancer, microbiome, immunotherapy, tumor stroma, antibiotics

## Abstract

Emerging research has uncovered the significance of microbiota in carcinogenesis, with specific bacterial infectious agents linked to around 15% of malignant tumors. This review is focused on the resident kidney microbiome, its composition, and alterations in various diseases. Recent studies have shown that bacteria can infiltrate the kidney, with differences between normal and tumor tissue. These studies have identified distinctive microorganisms unique to both conditions, hinting at their potential clinical relevance. Research into the kidney microbiome diversity reveals differences in tumor tissue, with specific taxa associated with different histological types. Notably, the alpha diversity indices suggest variations in bacterial content between tumor and normal tissue, offering insights into potential diagnostic and prognostic use of these markers. Better studied is the impact of the gut microbiome on therapy efficacy in malignant kidney tumors. Antibiotics, which can alter the gut microbiome, have been linked to survival outcomes in patients receiving targeted therapy and immunotherapy. The findings suggest that the uncontrolled use of antibiotics may not only contribute to bacterial resistance but also disrupt the normal microbiome, potentially influencing the development of oncological diseases. In-depth investigation into the resident kidney microbiome is essential for addressing fundamental and practical aspects of kidney tumor development.

## Introduction

As of today, it is considered that the human microbiota comprises approximately 1800 genera and over 40,000 bacterial strains [1]. In addition to bacteria, the human microbiota also includes archaea, eukaryotes such as protists, fungi, and nematodes, as well as viruses. The composition of the human microbiota is quite specific and individualized. At the same time, some changes in the microbiota can be observed as the body ages and in response to various external factors. In 2012, C. de Martel and others demonstrated that approximately 15% of human malignant tumors are associated with specific bacterial infectious agents [2]. The International Agency for Research on Cancer (IARC) identifies 10 microorganisms whose role in carcinogenesis has been established and proven [3]. The contribution of the gut microbiome to the development and progression of malignant tumors has been well studied. The role of the intestinal microbiota in response to chemotherapy and immunotherapy by regulating its effectiveness and side effects has also been described. Despite the significant research on the resident tissue microbiome in various organs in both healthy and pathological conditions, the kidney microbiome remains relatively poorly characterized. A substantial amount of research is dedicated to studying the influence of the gut microbiome in the development of various kidney pathologies. This review focuses on the resident kidney microbiome and its alterations in various diseases.

## Resident Renal Microbiome in Norm and Pathology

Until recently, it was believed that healthy kidney tissue was sterile and did not contain microorganisms. However, it has been discovered that in some diseases, bacteria can penetrate kidney tissue through the bloodstream [4]. The resident microbiome of kidney tumors is virtually undescribed.

The first study dedicated to the resident microbiome of the kidney was published in 2019 [5]. On a small sample of patients, the authors demonstrated that the number of microorganisms in conditionally normal kidney tissue is lower than in tumor tissue. The authors identified 3 domains, 15 phyla, 16 classes, 19 orders, 27 families, 28 genera, and 30 species of microorganisms. Additionally, this study identified microorganisms that are specific only to normal kidney tissue, such as the genera *Microbacterium, Pelomonas, Staphylococcus, Streptococcus, Leuconostoc garlicum*, and the species *Corynebacterium vitaeruminis, Anaerococcus nagyae, Ethanoligenens harbinense, Neisseria bacilliformis, Thermicanus aegyptius*, and *L. mesenteroides*, as well as species specific to tumor tissue, such as *Cyanophora paradoxa, Spirosoma navajo, Phaeocystis antarctica, Euglena mutabilis*, and *Mycoplasma vulturii*. The content of bacteria *Aeromonas salmonicida, Pseudoalteromonas haloplanktis, Parageobacillus toebii, Trachelomonas volvocinopsis, M. mycoides*, and *Halomicrobium mukohataei* significantly differed between tumor and normal kidney tissue [5].

Recently, another study focused on the resident microbiome of the kidney highlighted that that the alpha diversity indices Chao1, Ace, and Shannon do not differ between conditionally normal and tumor tissue, while a significant decrease in the Simpson index was observed in tumors [6]. It is worth noting that the Shannon index characterizes the diversity and evenness in the community structure, whereas the Simpson index indicates the degree of dominance of certain species in the community structure. Thus, the Shannon index is more sensitive to changes in the abundance of rare species, while the Simpson index is more sensitive to changes in the abundance of the most common species. The dominant types of bacteria present in tumor and conditionally normal tissue are *Proteobacteria* and *Firmicutes*. Significant differences in the relative abundance were observed only at the order level for *Burkholderiales* and the family *Comamonadaceae* between the studied sample groups. Furthermore, 10 taxa of microorganisms found in most of the studied samples were selected by the authors for further analysis. ROC analysis showed that a decrease in the relative abundance of *Klebsiella* (AUC = 0.86, *p* < 0.0001)*, Chloroplast* (AUC = 0.91, *p* < 0.0001), and *Streptophyta* (AUC = 0.89, *p* < 0.0001) allowed for differentiation between tumor and normal tissue with Chloroplast showing highest sensitivity of 91,67% and specificity of 83,33% [6]. For *Deinococcus* and *Phyllobacterium*, an increase in their content in the tumor also indicated renal cell carcinoma with moderate strength. Using Phylogenetic Investigation of Communities by Reconstruction of Unobserved States (PICRUSt) analysis, the authors identified differences between tumor and normal tissue in 9 KEGG pathways. Among them, 3 pathways (membrane transport, transcription, cell growth and death) were widely represented in tumor tissue, while 6 pathways (cellular mobility, signal transduction, metabolism, cofactor and vitamin metabolism, energy metabolism, and the endocrine system) were predominant in conditionally normal tissue [6]. It is known that bacteria can contribute to tumor development by activation of various signaling pathways. For example *Escherichia coli* induces DNA damage *in vivo* and triggers genomic instability in epithelial mammalian cells [7], *H. pylori* and *Salmonella typhi* activate β-catenin signaling, driving gastric and colon cancer respectively [8, 9] and *Prevotella, Streptococcus* and *Veillonella*, *in vitro* and *in vivo*, lead to PI3K and AKT signaling activation [10]. We have established that, the highest content of bacteria of the genus *Phyllobacterium* is observed in the case of papillary carcinoma, and their relative abundance of more than 1% tends to indicate a favorable prognosis of the disease [11].

In 2020, another study was published that conducted research on the microbiome of tumor and conditionally normal kidney tissue, as well as tumor thrombi in a sample of 6 patients. The authors noted significant changes in the alpha diversity of the examined samples, with the highest values of this parameter observed in tumor tissue. It was found that *Micrococcus luteus*, *Fusobacterium nucleatum*, *Streptococcus agalactiae*, and *Corynebacterium diphtheriae* are the most abundant species in tumor samples compared to the adjacent normal kidney and tumor thrombus [12].

Recently our group characterized the microbiome of kidney tumors of different histological types. No prior research had not yet been conducted on the microbiome of papillary and chromophobe kidney cancer. Our analysis of the taxonomic composition of kidney tissue’s microbial community revealed the presence of 14 phyla and 170 genera. The predominant types of microorganisms found in both tumors and samples from normal tissue were *Actinobacteria, Proteobacteria*, and *Firmicutes*. It was noted that normal kidney tissue had a higher number of microorganism taxa compared to tumors. Additionally, differences between normal kidney tissue and tumors of different histotypes were observed at the phylum level. For instance, bacteria of the *Tenericutes* type were present in ccRCC and papRCC tumors but absent in normal tissue and chromophobe tumors. Furthermore, bacteria phyla *Gemmatimonadetes, Chloroflexi, Fusobacteria, Parcubacteria*, and *Verrucomicrobia* were found only in samples from normal kidney tissue [13]. Analysis of alpha diversity and overall bacterial composition revealed significant differences. Tumor tissue exhibited lower diversity and bacterial content. The lowest number of bacteria was detected in the group of papillary tumors. A detailed analysis showed that, in general, kidney tissue was dominated by gram-positive microorganisms, except for clear cell carcinoma, in which gram-negative bacteria were dominant. No significant qualitative or quantitative changes in the microbiome were identified as the tumor progressed. However, there was a trend towards decreased bacterial load with disease progression [13]. These findings partially disagree with the results obtained by Wang and others [6]. This discrepancy may be attributed to the fact that our study used normal kidney tissue from patients without kidney tumor pathology as a control, rather than conditionally normal kidney tissue from renal cell carcinoma patients. Additionally, our study highlighted that the analysis of overall bacterial load did not have prognostic significance in the renal cell carcinoma group, while the number of identified microorganism taxa was a significant prognostic factor for the clear cell carcinoma variant of tumors [13].

Consequently, analyzing the resident microbiome associated with malignant kidney tumors of various histological types is important. This analysis will help to identify specific microorganisms and their combinations that have clinical and prognostic significance for each tumor type.

## Urinary Microbiome

Accumulating evidence from various studies indicate the association between the urinary microbiome and various kidney diseases. For example, it has been shown that the composition of the urinary microbiome changes in cases of acute kidney injury in both renal transplant and non-transplant patients [14]. It’s worth noting that urine was traditionally considered sterile. However, researchers have recently begun to challenge this theory. Rosalind Maskell, for instance, suggested that urine might contain slowly growing microorganisms that are not detectable using standard cultivation techniques [15]. With the advancement of genetic technologies and metagenomic sequencing, it has become evident that the urine of a healthy individual is not sterile [16, 17]. Recent studies indicate that the urinary tract harbors a large number of microorganisms [18], and changes in their composition occur in various diseases, including overactive bladder syndrome [19], urinary incontinence, interstitial cystitis [20], neurogenic bladder [21], sexually transmitted infections [16], and chronic prostatitis [22]. Though there are quite a few published studies of urine microbiome in bladder cancer patients [23] there is not much published on the urine microbiome in renal cell carcinoma patients. Hyun Kyu Ahn et al., demonstrated that there is no difference in alpha- and beta-diversity between renal cell carcinoma patients and healthy individuals. They also identified 8 species, the abundance of which differs significantly in renal cell carcinoma patients, with 6 of these—*C. acnes*, *P. lacydonensis*, *Micrococcus spp*., *C. granulosum*, *Tessaracoccus arenae*, and *Staphylococcus epidermidis* were more abundant in the renal cell carcinoma group [24].

Another study compared the urinary microbiome of renal cancer patients and patients with renal cyst. It was found that microbial diversity was significantly decreased in the clear cell RCC group, and two genera, *Gardnerella* and *Enterococcus,* were found to be dominant in the ccRCC group [25]. However, the microorganisms detected in urine may have different origins, including contamination from the prostate gland or the female urogenital tract microbiome, making it challenging to use urine analysis for the differential diagnosis of various conditions, especially oncological pathologies.

## Role of Antibiotics

Although our understanding of the kidney’s resident microbiome remains limited, extensive research has focused on exploring how the gut microbiome influences various diseases, including its role in kidney tumor development. In the final part of this review, we will focus on the influence of the gut microbiome on the effectiveness of different therapies for malignant kidney tumors. Modern targeted therapy and immunotherapy have made significant breakthroughs in the treatment of renal cell carcinoma (RCC) and are often used as first-line treatments. Unfortunately, all forms of such therapy are associated with side effects of various degrees, including asthenia, diarrhea, cardiac and skin toxicities, among others. Some studies suggest that part of these side effects may be mediated by changes in the composition of the gut microbiome, specifically an increase in bacteria of *Bacteroides* genus and a decrease in bacteria of *Prevotella* genus, resulting in intestinal damage [26]. Hahn and others conducted a study demonstrating that the use of antibiotics, which reduce the abundance of *Bacteroides* genus bacteria in the gut, significantly improved the survival of patients with metastatic RCC [27]. Similar findings were obtained when examining the impact of antibiotics on the outcome of immunotherapy. In patients with advanced RCC who received antibiotic therapy, a decrease in the frequency of objective responses to immune checkpoint inhibitors and progression-free survival was observed, which was not observed in patients receiving mTOR inhibitor therapy [28]. Similar results were reported by Derosa et al. in 2020, showing reduced overall and progression-free survival of RCC patients who received antibiotic treatment [29]. In their subsequent study, the authors demonstrated that an elevated level of specific bacteria (*Akkermansia muciniphila, Bacteroides salyersiae*, and *Eubacterium siraeum*) was associated with better survival in RCC patients and may enhance the efficacy of immunotherapy [30]. Overall, the link between the gut microbiome composition and the efficacy of immunotherapy is well-established. However, despite numerous studies, the precise mechanisms through which the microbiome exerts its influence remain unclear, and these studies often have significant limitations.

It’s worth mentioning a single study that focused on the analysis of the resident microbiome of kidney tumors and its association with the response to immunotherapy. In a sample of 22 patients, it was shown that tumors responding to immunotherapy were characterized by an enrichment of bacterial species such as *Bacillus thuringiensis, Comamonas testosteroni, Colletotrichum higginsianum*, and *Elaeis guineensis*. On the other hand, tumors in patients who did not respond to checkpoint inhibitor therapy exhibited enrichment of species including *Candidatus Promineofilum breve*, *Clostridioides difficile, Nocardia cyriacigeorgica, Streptomyces* sp. *CdTB01*, and *Streptomyces venezuelae* [31]. Based on these results, it can be speculated that the composition of the tumor microbiome may predict the effectiveness of therapeutic responses, alongside currently used markers.

## Discussion

Considering the growing interest to kidney microbiome and its potential implications for renal pathology, it is important to underline the existing gaps and limitations within this field. The complexity of the microbiome, characterized by its inter-individual variability due to diet, lifestyle, genetics, and environment‐poses a significant challenge in identifying universally applicable microbial biomarkers for kidney cancer or for predicting therapeutic outcomes.

Furthermore, there is a certain degree of methodological heterogeneity, encompassing variances in the approaches to sample collection, DNA extraction, and sequencing methodologies in the field. Lack of consistency in the research methods in studying kidney microbiome has led to discrepancies in findings, thereby hindering the ability to draw consistent conclusions across different studies. The establishment of standardized protocols is thus a critical need, aimed at enhancing the comparability and reproducibility of microbiome research findings.

Additionally, the bulk of the research has mainly employed cross-sectional study designs, which, although informative, capture merely a static picture of the microbiome. This approach largely overlooks the dynamic nature of the microbiome and its interaction with the progression of renal diseases, the impact of therapeutic interventions, and the direction of patient outcomes over time. The lack of longitudinal studies in this area underscores a significant knowledge gap, making future investigations necessary that can elucidate the temporal dynamics of the kidney microbiome in relation to renal health and disease. Moreover, despite the identification of associations between specific microbial signatures and kidney cancer, the causal mechanisms underlying these relationships remain largely unexplored. Experimental studies using animal models or *in vitro* systems are needed to unravel the causative links between particular microbes or microbial consortia and the pathogenesis of kidney cancer.

In conclusion, while the study of the resident kidney microbiome is a promising direction for novel diagnostic and therapeutic developments in renal disease, especially cancer, it is linked to notable challenges. Addressing these limitations through the adoption of standardized research methodologies, the expansion of mechanistic studies, and the consideration of inter-individual variability will be important in advancing our understanding of the full potential of microbiome research in the field of renal medicine.
